# Evaluation of ranges of motion of a new constrained acetabular prosthesis for canine total hip replacement

**DOI:** 10.1186/1475-925X-12-116

**Published:** 2013-11-10

**Authors:** Ching-Ho Wu, Cheng-Chung Lin, Tung-Wu Lu, Sheng-Mao Hou, Chih-Chung Hu, Lih-Seng Yeh

**Affiliations:** 1 Institute of Veterinary Medicine, National Taiwan University, Taipei, Taiwan; 2 Department of Small Animal Surgery, National Taiwan University Veterinary Hospital, Taipei, Taiwan; 3 Institute of Biomedical Engineering, National Taiwan University, Taipei, Taiwan; 4 Department of Orthopedic Surgery, School of Medicine, National Taiwan University, Taipei, Taiwan; 5 Shin Kong Wu Ho-Su Memorial Hospital, Taipei, Taiwan; 6 Department of Mechanical Engineering, Ming Chi University of Technology, Taipei, Taiwan

**Keywords:** Total hip replacement, Constrained acetabular component, Range of motion, Computer simulation, Canine

## Abstract

**Background:**

Total hip replacement (THR) is considered to be the most effective treatment option for advanced osteoarthritis of the hip in large breed dogs. However, a proportion of post-THR patients suffer prosthesis dislocation for various reasons, which may be addressed by a constrained acetabular prosthesis design. The study proposed a new THR with constrained acetabular component that aimed to decrease the incidence of postoperative dislocation while maintaining the necessary range of motion (ROM); and, through computer-simulated implantations, evaluated the ROM of the THR with and without malpositioning of the acetabular component.

**Methods:**

A new THR with a constrained acetabular component that had an inward eccentric lining and a 60° cut-out on the dorsal side was designed, and its computer-aided design models were implanted into the pelvic and femoral models reconstructed from the computed tomography data of six healthy Labrador Retriever dogs. The allowable and functional ROM of the implanted THR were determined via computer simulations. The contact patterns between the bone or the prosthetic components at extreme positions of the THR were analyzed. Influence of malpositioning of the acetabular component on the ROM was assessed.

**Results:**

The means (SD) of the functional ranges for flexion, extension, adduction, abduction, internal rotation and external rotation were 51.8° (6.6°), 163.3° (7.3°), 33.5° (5.7°), 74.0° (3.7°), 41.5° (8.3°) and 65.2° (9.9°), respectively. Malpositioning of the acetabular component by 20° in one direction was found to reduce ROM in other directions (reducing lateral opening: flexion: 12°, adduction: 20°, internal/external rotations: < 20°; increasing lateral opening: extension and abduction: < 16°; reducing retroversion: extension: < 20°, abduction: 15°, external rotation: < 20°; increasing retroversion: flexion: < 20°, abduction, adduction and internal rotation: 20°).

**Conclusions:**

From the computer-aided surgical simulations, the new THR was found to have sufficient functional ranges for flexion, extension, abduction, adduction and external rotation for Labrador Retrievers. Analysis of the malpositioning of the acetabular component suggests that accurate placement of the acetabular component is critical for achieving desirable ROM for daily activities.

## Background

Total hip replacement (THR) is considered to be the most effective treatment option for advanced osteoarthritis of the hip in large breed dogs, primarily subsequent to dysplasia of the joint and other disabling conditions. Previous studies have shown that it is effective in pain relief and restoration of the joint function [1-4]. However, the long-term survival rate of the prosthesis can be affected by complications [5-9], including femoral fracture [8], osteolysis [10,11], osteopenia [12] and luxation of prosthetic components associated with the malpositioning of the acetabular component [6,7,13].

Among the postoperative complications, prosthesis dislocation has a severe impact on the function of the joint and can lead to the final failure of the primary THR, requiring revision surgery. With the existing unconstrained acetabular prosthesis design, it has been reported that as high as 4.7% to 11% of canine post-THR patients suffered prosthesis dislocation [6,9,14]. Several factors affect the occurrence of THR luxation, including malpositioning of the acetabular component [6], insufficient length of the femoral neck [15], preexisting subluxation [16], impingement between components or bones and the difficulty of getting these patients to follow instructions for restricted activities and gradual rehabilitation, etc. It appears that a THR design with reinforced articulation between the femoral head and the acetabular cup, which is less sensitive to the malpositioning of the cup, may be helpful for reducing the risk of luxation of THR and thus improving its long-term success.

The problem of postoperative dislocation has been investigated in human THR, which may lend useful information to the resolution of the same problem in the design of canine THR. An efficient approach to reducing the incidence of dislocation is a constrained acetabular prosthesis design, the lining of which covers more than half of the surface area of the femoral head [17]. The constrained acetabular component has often been used in revision surgery to minimize intraoperative instability and postoperative dislocation of THR [18,19]. It is also employed occasionally in primary surgery for patients with neuromuscular insufficiency. However, a major limitation of the existing constrained acetabular design is the reduced range of motion (ROM) [20], leading to the potential risk of impingement, component failure, and the final dislocation and implant loosening. With the reduced ROM provided by the constrained design, the correspondence of this allowable ROM and the desired functional ROM becomes even more critical, requiring accurate positioning of the prosthesis components relative to the underlying bones [6,13]. A malpositioned acetabular component may have a significant impact on the unrestricted functional ROM of the replaced joint, which should be considered during the design of the prosthesis, especially for canine patients who are less likely to control their activity so as to avoid extreme joint positions and the risk of dislocation.

The purposes of this study were to propose a new THR with constrained acetabular cup design for dogs which aims to decrease the incidence of postoperative dislocation while maintaining the necessary ROM by adopting an inward eccentric lining and a partial cut-out on the dorsal side of the acetabular component; and, through computer-simulated implantations, to evaluate the ROM of the THR in a group of Labrador Retriever dogs with and without malpositioning of the acetabular component.

## Methods

### Total hip design with a constrained acetabular component

**Figure 1:**
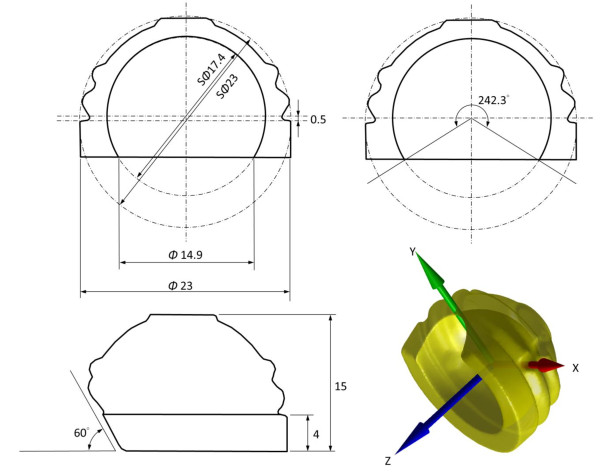
**Computer-aided model of the acetabular component: the cross-sectional dimensions and the 3D computer model of the acetabular component of the new THR. (Units: mm and degrees).** An acetabular prosthesis coordinate system (APCS) of the acetabular component was defined with three orthogonal axes. The Z-axis of the APCS was normal to the ventral opening surface, the X-axis was parallel to a line connecting the mid-points of the junctions between the ventral and dorsal opening surfaces, and the Y-axis was obtained as the cross-product of the Z-axis and X-axis.

A new THR with a constrained acetabular cup design with an eccentric inner surface was developed (Figure 1). The acetabular component was designed as a unitary component with an outer spherical surface of 23 mm in diameter and an inner spherical surface of 17.4 mm in diameter, giving a femoral head coverage angle of 242.3 degrees. A coverage angle of greater than 180 degrees provided a restraining force preventing the femoral head from dislocating from the cup. However, too much coverage angle would increase the height of the cup, increasing the bone resection needed for implantation. To maintain good stability while minimizing bone resection, the current design adopted an eccentric outer and inner surface design. The inner surface was shifted towards the top of the component by 0.5 mm, increasing the coverage of the femoral head by 7.6 degrees when compared to the same design without eccentric shift. The final design gave an inner surface opening of 14.9 mm in diameter, enabling the acceptance of a femoral head of 17 mm in diameter that was fitted to a tapered femoral stem. With these features, the acetabular coverage would surpass the equator of the femoral head, enabling the femoral head to be snapped into the acetabular component with increased coverage. Given the inherent constrained geometry, the restricting force of the constrained design was expected to be larger than that of a non-constrained design, which would help reduce the potential risk of dislocation after THR. In order for a larger range of abduction for the necessary ROM for daily activities of canines, a partial cut-away region of 60° relative to the opening surface was made on the dorsal rim of the acetabular component at the 12 o’clock position (Figure 1). This cut-away was chosen to be consistent with the natural morphology of the dorsal rim of the canine acetabulum [21], which would help minimize the possible impingement between the acetabular component and the femoral neck.

A prototype of the current THR design was manufactured with the acetabular cup made of high-density polyethylene as a unitary component and the femoral component made of titanium alloy. The prototype was tested for its mechanical ROM, defined as the maximum rotation of the femoral component within the acetabular component with respect to the coordinate system embedded to the acetabular component called the acetabular prosthesis coordinate system (APCS), without being implanted in a dog. The Z-axis of the APCS was normal to the ventral opening surface, the X-axis was parallel to a line connecting the mid-points of the junctions between the ventral and dorsal opening surfaces, and the Y-axis was obtained as the cross-product of the Z-axis and X-axis (Figure 1). The maximum rotation of the femoral component relative to the acetabular component was measured using infrared stereophotogrammetry (VICON, OMG, UK). For this purpose, three infrared retroreflective markers (diameter = 3 mm) each were attached to the acetabular and femoral components, and the key landmarks necessary for the definition of the APCS were determined relative to the markers using a pointer which was also visible to the stereophotogrammetric system. This allowed the measurement of the positions of the key landmarks in space by tracking the markers on the components. The femoral component was moved manually to perform maximum circumduction, with continuous contact of the femoral neck on the acetabular component, and rotation about the Z-axis while the marker trajectories were measured simultaneously. The measured data were then used to calculate the mechanical ROM about the three axes of the APCS using simple algebraic manipulations.

### Subjects

Six healthy client-owned Labrador Retriever dogs (age: 3.6 ± 1.5 years; body mass: 27.4 ± 3.9 kg; body height: 57.3 ± 2.0 cm; body length: 65.5 ± 2.4 cm; four males and two females) without any neuromusculoskeletal diseases at the hip joints participated in the current study, with written informed consents from the owners as approved by the Institutional Animal Care and Use Committee. The subjects were recruited from different families (different owners) and were not genetically related within 5 generations. Body height, defined as the distance between the level ground and the top of the shoulder while the animal was standing erect in the neutral position, was measured using a tape measure. Similarly, body length was defined and measured as the distance between the front of the chest and the base of the tail. The pelvis and the femur of the dogs were scanned by computed tomography (CT, Somatom Sensation16, Siemens Medical Systems, Germany) from the cranial border of the ilium to the mid line of the knee joint with a CT slice thickness of 0.7 mm, a pixel size of 0.725 mm × 0.725 mm and an averaged dose length product (DLP) of 154 mGycm. During the CT data collection, the dogs were maintained anesthetically using intravenous propofol and were positioned in dorsal recumbency with the hind limbs caudally extended. From the CT data the bony parts, namely the pelvis and right femur, were semi-automatically segmented from all image slices to obtain subject-specific surface models of the bones using a commercially-available software package (Amira, Visage Imaging Inc., Germany).

### Computer-simulated implantation

**Figure 2:**
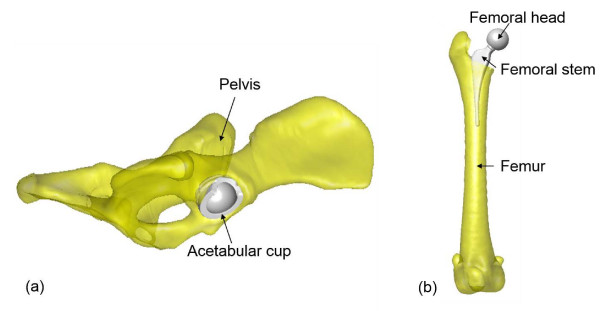
Computer simulation of THR surgery using computer models of the patient-specific bones and THR models: (a) implantation of the acetabular component (grey) into the pelvic bone (yellow), and (b) implantation of the femoral component (grey) into the femur (yellow).

With the subject-specific models of the bones, computer-simulated implantations of the new total hip were performed with the computer-aided design (CAD) models of the prosthesis components, namely the acetabular component, femoral head and the femoral stem, via a commercial reverse engineering software package (Geomagic Studio, Geomagic, Morrisville, NC, USA). With the assistance of the graphic user interface of the software, a senior surgeon (WCH) incorporated the acetabular component of the THR into the right pelvis, replacing the natural acetabulum to simulate the standard surgical procedure [22] (Figure 2a). The acetabular component was positioned such that its ventral opening surface was aligned with the best-fitted plane of the acetabular ventral rim to match with the native orientation, and such that the dorsal edge of the component was aligned with the dorsal rim of the acetabulum of the dog, giving the best angles of the lateral opening and the retroversion of the acetabular component [21]. The position of the acetabular component along the X-axis of the APCS was determined such that the mid-point of the line connecting the two junctions of the dorsal and ventral opening surfaces of the acetabular component matched that of the acetabular bone (Figure 2a). The angular position of the acetabular component about the Z-axis (normal to the ventral opening surface) was determined by aligning the junctions of the dorsal and ventral opening surfaces of the acetabular component to those of the dorsal and ventral rims of the acetabular bone (Figure 2a). The model of the femoral stem was incorporated into the right femur after the resection of the femoral head and neck as in routine real-life THR surgery. The femoral head was fitted to the femoral stem according to the requirements of the modular design (Figure 2b).

### Bone-embedded anatomical coordinate systems

**Figure 3:**
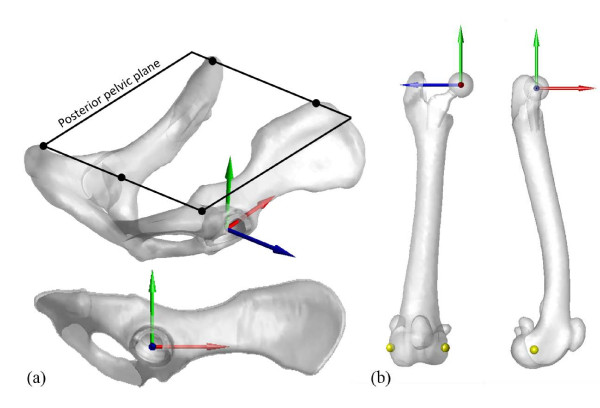
**Anatomical coordinate system (ACS) of bones: the ACS of the pelvis (a) and femur (b).** The positive X-axis (red) indicates the anterior direction, the positive Y-axis (green) indicates the dorsal direction, and the positive Z-axis (blue) is directed to the right. The landmarks on the epicondyles used for defining the Z-axis are marked in yellow.

For the description of the motion of the hip, each model pelvis and femur was embedded with an anatomical coordinate system (ACS) (Figure 3). For the pelvic ACS, a reference plane called the posterior pelvic plane (PPP) was first defined as the plane containing the dorsal tops of the iliac crests and the dorsal tops of the ischial tuberosities [21,23]. The normal vector of the PPP was then defined as the Y-axis. The medial-lateral axis (Z-axis) was defined as the line connecting the dorsal tops of the iliac crests. The anterior-posterior axis (X-axis) was determined as the axis perpendicular to both the Y- and Z-axes [21]. For the femoral ACS, the medial-lateral axis (Z-axis) was defined as the line connecting the lateral and medial epicondyles, directed to the right. The X-axis was defined as the axis normal to the plane intersecting the epicondyles and greater trochanter, directed anteriorly. The Y-axis was determined as the axis perpendicular to both the X- and Z-axes [24]. The origins of the pelvic and femoral ACS were defined at the coincident centers of the acetabulum and femoral head, respectively. The neutral position of the hip was defined as the pose when the pelvic ACS coincided with the femoral ACS. All anatomical landmarks were selected manually by the senior surgeon (WCH) from the corresponding surface models using Geomagic (Geomagic Studio, Geomagic, Morrisville, NC, USA).

### Range of motion analysis

**Figure 4:**
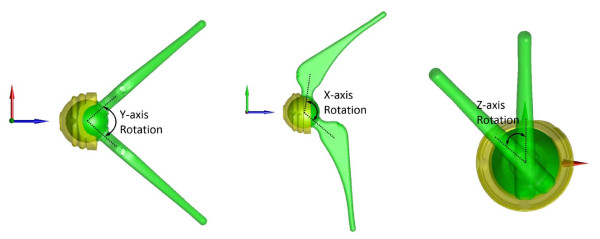
Definition of the mechanical ROM of the new constrained THR.

**Figure 5:**
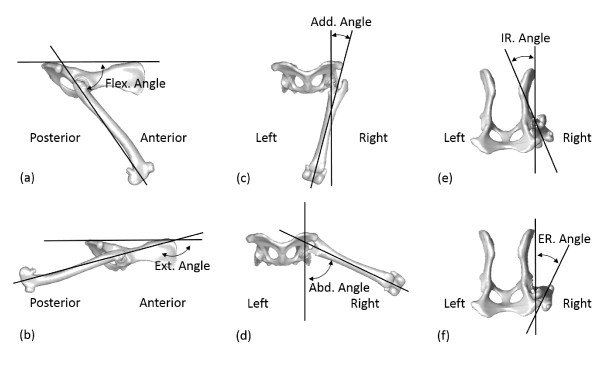
**Definitions of the allowable and functional ROM of the new THR after simulated implantation.** The allowable ROM was determined as the maximum angles of (**a**) flexion, (**b**) extension, (**c**) adduction, (**d**) abduction, and (**e**) internal and (**f**) external rotation of the femoral component within the acetabular component relative to the pelvic ACS without constraints or limitations from the bony structures. The functional ROM was those angles allowed by constraints from both the implants and the bony structures.

Three types of ROM were determined for the new THR: mechanical, allowable and functional ROM. The mechanical ROM of the THR indicated the maximum angles of the three rotations of the femoral component within the acetabular component outside the body, and was determined with respect to the APCS (Figure 4). The other two types of ROM were related to the THR after simulated implantation. The allowable ROM of the THR was determined as the maximum angles of the flexion/extension, abduction/adduction and internal/external rotations of the femoral component within the acetabular component relative to the pelvic ACS without constraints or limitations from the bony structures, while the functional ROM was those allowed by both the implants and the bony structures (Figure 5). Effects of the soft tissues around the hip were not included in the current assessment. For the calculation of the allowable and functional ROM, in terms of the maximum angles of the femoral ACS (embedded into the femur/femoral component model) relative to the pelvic ACS (embedded into the pelvis/acetabular component model), an automatic computerized procedure was developed and implemented as a self-developed program in Matlab (Mathworks, Inc., USA) on a PC. The procedure started from the neutral position at which the femoral and pelvic ACS coincided with each other. The assembled model of the femur/femoral components represented by the femoral ACS was then rotated about its origin about the three anatomical axes of the pelvis ACS separately at increments of 1°. The rotation continued until the femur/femoral component model first collided with the pelvis/acetabular component model (either bone-to-bone, component-to-component, or bone-to-component) as detected using a point-in-polyhedron test [25]. The hip joint angles at this extreme position were then recorded as the functional ROM of the THR. The same procedures were then repeated without constraints from the bony structures (i.e., only component-to-component collision would be detected) to determine the allowable ROM. Note that since the flexion angle was defined as the angle between the longitudinal axis of the femur and the PPP according to the common definition in the relevant literature, the range of flexion actually increased when the value of the flexion decreased (Figure 5).

In order to assess the effects of the malpositioning of the acetabular component on the functional ROM of the THR, the acetabular component was systematically deviated from its neutral orientation simulating common positional errors during surgery, namely rotational deviations in the lateral opening and retroversion. The simulated malpositions ranged from -20° to 20° at increments of 5°, which were based on the possible range of mal-alignment during surgery reported in the literature [6]. For each simulated orientation of the acetabular component, the functional ROM of the THR was determined using the above-described automatic procedure.

## Results

The mechanical ROM of the THR obtained from the computer models were 117.3°, 81.8° and 360° for rotations about the X-axis, Y-axis and Z-axis, respectively. The results agreed well with those measured from the prototype with only small differences in the X-axis and Y-axis rotations, which were less than 1.9° and 1.8°, respectively. The allowable and functional ranges of flexion, adduction and internal rotation were identical, while the allowable ranges of extension, abduction and external rotation were slightly greater than the corresponding functional ROM (Table 1). The ensemble-averaged functional ROM in the sagittal, transverse and horizontal planes were 111.5 ± 5.2°, 107.5 ± 4.9° and 106.7 ± 7.1°, respectively (Table 2).

**Table 1:** The means, standard deviations (SD), minimum (Min) and maximum (Max) values of the functional ranges of motion and allowable ranges of motion of each of the rotational components across all subjects

	**Rotations**	**Mean**	**SD**	**Min**	**Max**
Functional	Flexion	51.8	6.6	44	61
	Extension	163.3	7.3	157	177
	Adduction	33.5	5.7	25	39
	Abduction	74.0	3.7	70	80
	I. Rotation	41.5	8.3	28	49
	E. Rotation	65.2	9.9	51	76
Allowable	Flexion	51.8	6.6	44	61
	Extension	164.2	7.6	157	177
	Adduction	33.5	5.7	25	39
	Abduction	79.8	7.2	73	92
	I. Rotation	41.5	8.3	28	49
	E. Rotation	69.5	13.0	51	88

(Unit: degree).

**Table 2:** Functional ranges of motion of the rotation components of the hip and the overall ranges of motion in the three anatomical planes from the current and previous studies

	**Flex (°)**	**Ext (°)**	**Add (°)**	**Abd (°)**	**IR (°)**	**ER (°)**	**Sagittal (°)**	**Transverse (°)**	**Horizontal (°)**
Current study (Functional ROM)	51.8	163.3	33.5	74.0	41.5	65.2	111.5	107.5	106.7
Mann et al. [30] (mix-breed dogs)	46	164	27	85	55	50	118	112	105
Thomas et al. [28] (German Shepherds)	44	155	-	-	-	-	111	-	-
Jaegger et al. [27] (Labrador Retrievers)	50	162	-	-	-	-	112	-	-
Lin et al. [29] (mix-breed dogs)	54.9	149.8	28.5	82.8	42.1	61.4	94.9	111.3	103.5

Flex: flexion; Ext: extension; Add: adduction; Abd: abduction; IR: internal rotation; ER: external rotation.

**Figure 6:**
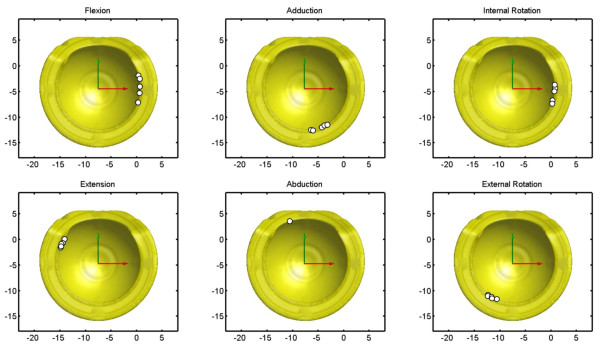
**Contact pattern of the new THR: the contact positions of the femoral component on the acetabular component (white dots) for six dogs at six extreme positions of the femur with respect to the pelvis (i.e., maximum flexion, extension, adduction, abduction, and internal and external rotation).** Each white dot represents the contact point of a dog.

The collision points on the acetabular component by either the femoral bone or the prosthetic component were found to be predominately on the anterior side (around the 3 o’clock position) for flexion and internal rotation, and around the 5 and 7 o’clock positions on the ventral side for adduction and external rotation, respectively (Figure 6). Those for extension were found to be located on the posterior and dorsal sides (10 o’clock position). The limitation on the functional range of abduction was mainly from bone-to-bone (femur-to-pelvis) collisions, and the positions varied between dogs, being at the cutaway region of the acetabular component in one dog, and on the pelvic bones in the others (Figure 6).

**Figure 7:**
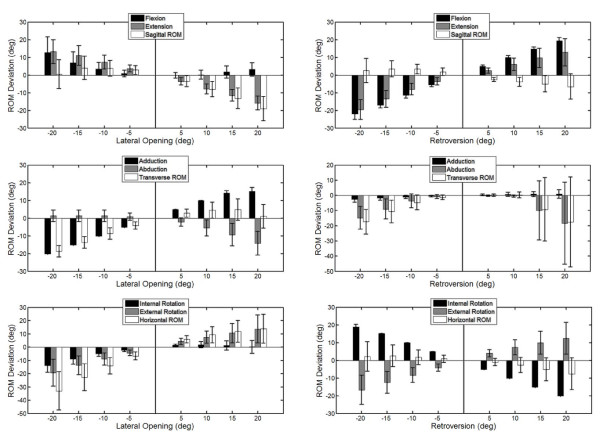
Influence of component malpositioning: influence of the malpositioning of the acetabular component in the lateral opening and retroversion on the functional ROM in the three anatomical planes.

With decreasing lateral opening angles of the acetabular component, the ROM in the transverse and the horizontal planes was decreased, but slightly increased in the sagittal plane (Figure 7). In contrast, increased lateral opening angles were found to lead to decreased ROM in the sagittal plane but increased ROM in the other planes. Increased retroversion was found to decrease the ROM in all planes, while decreased retroversion was found to reduce the ROM in the transverse plane with slight effects in the other two planes (Figure 7).

## Discussion

The current study described a newly-developed THR that had a constrained acetabular component with an inward eccentric lining design and a 60° cutaway region on the dorsal side. The design was aimed to increase the coverage of the femoral head while retaining the necessary ROM for daily activities. With computer-aided surgical simulations, the new THR was found to have sufficient functional ranges for flexion, extension, abduction, adduction and external rotation for Labrador Retrievers and similar breeds. The associated bone/prosthesis contact patterns suggest that an additional cutaway region on the anterior side of the component may be helpful for further increasing the ranges for flexion and internal rotation. Analysis of the malpositioning of the acetabular component suggests that accurate placement of the acetabular component is critical for achieving desirable ROM for daily activates.

Computer simulations of THR implantation were used in the current study to assess the ROM of the new THR, which had several advantages over traditional methods such as measuring the ROM directly on living subjects using either goniometers [26-28] or photographic systems [29]. The primary advantage of computer simulation was that it was free from intra- and inter-observer variability, and thus produced high measurement reliability. The current approach enabled the measurement of both allowable and functional ROM considering direct constraints from the bones and implants, which was in contrast to previous skin-based measurements that involved errors associated with the overlying soft tissues [26-28]. Another advantage of the current approach was the opportunity to determine quantitatively the contact patterns between the components for further design improvements, and the option to study the effects of the malpositioned acetabular component on the functional ROM to guide future improvement of surgical implantation procedures.

The allowable and functional ROM measured in the current study provided useful data for the baseline (or maximum) ROM the new THR could achieve, although physiological ROM was not determined in the current study because of the difficulty in simulating soft tissues including the ligaments, joint capsules and muscles. In general, the functional ranges of the flexion, extension, adduction and external rotation of the current THR were comparable to those of the native hip reported in the literature (Table 2). In the sagittal plane, the functional ranges of flexion and extension of the proposed constrained THR were found to be well within the ranges of the native hip joint (Table 2), suggesting that the proposed constrained THR could provide the dogs with sufficient ROM in the sagittal plane. In the transverse plane, the ranges of abduction were about 10° smaller than the reported values for native hips from mix-breed dogs (Table 2). While the range of abduction appeared to be slightly smaller than the native ranges of dogs of mixed breeds, the range of adduction provided by the component was sufficient (Table 2). Furthermore, the fact that the ranges of abduction of five out of the six tested joints were determined by the bones instead of the prosthesis components (Figure 6) indicates that the proposed constrained THR with the current angle of the dorsal cutaway region, once implanted in the correct position, does not restrict the range of abduction necessary for the Labrador Retrievers in any activity of daily living. Nonetheless, there was a slight difference between the current range of abduction and those previously reported (Table 2).

For the ROM in the horizontal plane, the range of external rotation in the current study appeared to be well-sufficient for dogs (Table 2), but comparisons of the range of internal rotation with the literature showed different results because the reported values varied among studies. While the current results were comparable to one study involving Labrador Retrievers [29], an increased range of internal rotation of about 13° was reported in another study [30] (Table 2). These slight differences between the current values and those previously reported may be a result of breed variations or differences in the accuracy of measurement methods as discussed above. Further measurements of the THR in other breeds using the current approach may be needed to evaluate whether the ranges of abduction and internal rotation are sufficient for these breeds and whether modification is needed.

The positions of the contact between the acetabular and the femoral components occurred primarily on the opening surface of the acetabular component for all kinematic components except abduction (Figure 6). Without the design of the cutaway region of the acetabular component, contacts during abduction are likely to be on the dorsal region of the acetabular open surface, limiting the functional range of abduction and increasing the risk of dislocation or component failure. For flexion, extension, adduction and external rotation components, the proposed THR has been found to provide sufficient functional ranges of motion necessary for the Labrador Retrievers. The odds are small that collisions occur between the acetabular component and either the femoral bone or prosthetic component. A potential modification that can be made on the current design for minimizing component-collisions could be the increase of the allowable ranges of internal rotation. Since the contacts points for extreme internal rotation were on the anterior side of the acetabular component (3 o’clock position) (Figure 6), an additional cutaway region on the anterior side may be helpful to achieve the necessary ROM if so required.

Malpositioning of the acetabular component affected the ROM of the replaced hip. Deviations in the lateral opening or retroversion from the neutral position will affect the ROM of all motion components: some increased but others decreased. This was not unexpected because the ventral rim of the native acetabulum and the opening surface of the acetabular component were essentially 3D in shape and in space. Increased ROM in one anatomical plane will naturally reduce the ROM in one or more of the other components. The current analysis showed that any inaccuracy of up to 15 degrees of retroversion will reduce the range of abduction by about 10 degrees. Since an increase in the retroversion of the acetabular component will reduce the overall ROM in all anatomical planes, malpositioning with an excessive increase in retroversion should be avoided. In contrast, a decrease of 5° in retroversion (cranial rotation) may be beneficial for increasing the ranges of internal rotation and flexion with a slight compromise of ranges of extension and external rotation. This is helpful for improving the current THR design that had slightly smaller internal rotation but greater ranges of extension and external rotation. From the current results of the malpositioning analysis of the THR, it is suggested that the acetabular component be implanted according to the native orientation with a slight decrease in the retroversion to provide ranges of motions required by Labrador Retrievers. For this purpose, a well-designed surgical guiding device is critical for accurate component placement for optimizing the performance of the THR.

In the assessment of the functional ROM of the proposed constrained acetabular component, the current study was limited to healthy Labrador Retrievers. The acetabular component was placed with reference to the native orientation of the acetabular geometry of the individual dog. However, in practice the THR are implanted to treat degenerated or diseased hip joints with deformed anatomy and osteophytes, which may affect the accurate positioning of the cup following the current surgical guideline, and thus the final functional ROM. Therefore, further study on dogs with hip dysplasia or degeneration will be needed to test whether the functional ROM would be different from the current results. Further study is also needed to investigate the stress and wear behavior of the proposed constrained cup THR design as limited ROM has been associated with risks of increased stress between interfaces, accelerated wear and eventual component failure in human constrained THR [31].

## Conclusions

A total hip replacement having a constrained acetabular component with an inward eccentric lining design and a 60° cutaway region on the dorsal side was developed in the current study. With computer-aided surgical simulations, the new THR was found to have sufficient functional ranges for flexion, extension, abduction, adduction and external rotation for Labrador Retrievers. Further increases in ranges of flexion and internal rotation may be achieved by an additional cutaway region on the anterior side of the component, which may be helpful for its application in other breeds. Analysis of the malpositioning of the acetabular component suggests that accurate placement of the acetabular component is critical for achieving desirable ROM for daily activities. Further study should be conducted on the design of a surgical guiding device for accurate component placement for optimizing the performance of the THR.

## Abbreviations

THR: Total hip replacement; ROM: Range of motion; CT: Computed tomography; CAD: Computer-aided design; ACS: Anatomical coordinate system; APCS: Acetabular prosthesis coordinate system; PPP: Posterior pelvic plane; SD: Standard deviation.

## Competing interests

The authors declare that they have no competing interests.

## Authors’ contributions

C-HW carried out the studies of design and evaluation of the canine THR, participated in the CT data collection and data analysis, and drafted the manuscript. C-CL developed the software programs, performed data analysis and helped draft the manuscript. T-WL participated in the design of the THR, the methodological development, the interpretation of the results and the drafting the manuscript. S-MH participated in the design of the THR, CT imaging, and the revision of the manuscript. C-CH participated in data analysis, engineering drawing and manuscript drafting. L-SY participated in the design of the THR, methodological development and drafting of the manuscript. All authors read and approved the final manuscript.
